# Socioeconomic differences in cancer survival: The Norwegian Women and Cancer Study

**DOI:** 10.1186/1471-2458-9-178

**Published:** 2009-06-08

**Authors:** Tonje Braaten, Elisabete Weiderpass, Eiliv Lund

**Affiliations:** 1 Institute of Community Medicine, University of Tromsö, Tromsö, Norway; 2 Department of Medical Epidemiology, Karolinska Institutet, Stockholm, Sweden,; 3 The Cancer Registry of Norway, Oslo, Norway; 4 Samfundet Folkhälsan (NGO), Helsingfors, Finland

## Abstract

**Background:**

Cancer survival has been observed to be poorer in low socioeconomic groups, but the knowledge about the underlying causal factors is limited. The purpose of this study was to examine how cancer survival varies by socioeconomic status (SES) among women in Norway, and to identify factors that explain this variation. SES was measured by years of education and gross household income, respectively.

**Methods:**

We used data from The Norwegian Women and Cancer Study, a prospective cohort study including 91 814 women who responded to an extensive questionnaire between 1996 and 1998. A total of 3 899 incident cancer cases were diagnosed during follow-up, of whom 1 089 women died, 919 of them from cancer. Cox Proportional Hazards Model was used to calculate relative risks (RR) of mortality and 95% confidence intervals.

**Results:**

We observed an overall negative socioeconomic gradient in cancer survival, which was most evident in the site specific analyses for survival of ovarian cancer by years of education. For colorectal cancer, mortality increased with years of education, but not with income. After adjustment for household size, marital status, disease stage, and smoking status the SES variation in cancer survival became non-significant. We found that the unequal socioeconomic distribution of smoking status prior to diagnosis contributed considerably to the poorer survival in low SES groups.

**Conclusion:**

We found an overall negative socioeconomic gradient in cancer survival when SES is measured as years of education or gross household income. Smoking status prior to diagnosis was an important predictive factor for socioeconomic variation in survival.

## Background

The association between socioeconomic status (SES) and cancer survival has been examined by several epidemiologic studies within a variety of study designs. A number of these are ecologic studies using geographical area based measures as SES indicators (comparing richer with poorer areas). Others are hospital-based or record linkage cohort studies with individual information on socioeconomic status measured by socioeconomic group, income or level of education [1-4]. Occasionally, health insurance status has been applied as a proxy of SES [5,6]. One cohort study among men has been able to consider lifestyle factors such as smoking and alcohol consumption according to SES and cancer survival. Regardless of study design, a number of studies have found an improved cancer survival by increasing SES, both overall and for specific anatomic sites, especially for cancers of relatively good prognosis such as female breast, corpus uteri, and bladder cancer [7]. A few studies find no association between SES and overall cancer survival, whereas site-specific null associations are more frequently reported. In general, the observed SES differences in survival seem to be lower in ecologic studies than in studies with individual assessment of SES [8]. Tumour characteristics as stage of disease has been claimed to contribute to the SES variation in cancer survival, whereas the limited information on lifestyle factors in previous studies leaves the role of patient characteristics unclear [7].

We present here results from a prospective cohort study where we evaluated how socioeconomic conditions at time of recruitment affect the likelihood of cancer survival. We studied both overall survival, and survival for selected sites, using information from death certificates to identify the cancer deaths. The comprehensive information collected in The NOWAC Study enables us to assess individual's lifestyle before diagnosis besides tumour characteristics as potential confounding factors of socioeconomic variations in cancer survival.

## Methods

### The Norwegian Women and Cancer Study

The Norwegian Women and Cancer Study was initiated in 1991 as a prospective, population-based cohort study recruiting 57 600 women aged 34–49 years (response rate 57.6%) who answered a four pages questionnaire. In 1996 the cohort expanded further and 44 843 women (56.8% of the invited) aged 30–69 years were included by responding to an eight pages questionnaire. A similar questionnaire was mailed to the initial sub-sample in 1998, of whom 46 971 women (81.5%) responded. The present study population is constituted by the sub-sample enrolled in 1996 together with the responders of the second questionnaire in 1998, 91 814 women in total. The questionnaires as well as other details of the cohort can be found at . The validity of The NOWAC Study has been assessed previously [9].

### Follow-up

Follow-up was achieved through linkages of the cohort data set to national registers by the personal identification number. The cancer data was provided by the Cancer Registry of Norway, and information on death and emigration was collected from the Cause of Death Register and the Central Population Register of Norway. These registers are considered to be virtually complete.

Among an initial study population of 91 814 women aged 30–69 at recruitment, a total of 3 899 incident primary invasive cancer cases were diagnosed before 1 January 2005, of whom 1 089 women died, 919 of these from the cancer they were diagnosed during follow-up. We excluded 50 women without any information on adult SES, leaving 3 849 incident cancer cases. The participants of The NOWAC study have been asked about one or both of the SES measures, and thus the number of cases included vary between the two models. We have information on education for 3 603 women who developed cancer, and on income for 3 575 women. From each analysis of solid tumours we further excluded women with missing information on covariates included in the respective multivariate model. The follow-up started at the date of diagnosis and ended five years later (at the latest 31 December 2005), or at emigration or death, whichever occurred first.

### Classification of socioeconomic status

#### Education

In the questionnaire, women were asked the total number of years they attended school. The choice of classification is related to levels in the educational system in Norway. Compulsory school attendance increased from seven to nine years in 1965. Thus, 7–9 years of education means primary school with at most two years of additional education. Women with 10–12 years of education may have completed secondary school, or up to five years of professional training. Education lasting 13–16 years corresponds to a university bachelor degree, or, in some instances, several professional training sessions at a lower level. The highest category comprises women with more than 16 years of education, which mainly corresponds to a university master level.

#### Income

The women were asked for the gross household income per year given as five intervals equally spaced by each NOK 150 000 (approximately 17 500 EURO), with the highest category defined as more than NOK 600 000 (70 000 EURO).

### Statistical analysis

We applied Cox Proportional Hazards Models to perform the statistical analyses, using the SAS Software Package (version 9.1) to calculate hazard ratios of mortality with corresponding 95% confidence intervals. The hazard ratios are interpreted as estimates of the reported relative mortality risks (RR), and the term survival is used analogously to mortality risk.

The associations between cancer survival and SES were first examined in age adjusted analyses. Whenever a variation in risk by SES was observed, potential confounding variables were added stepwise to the models for all solid tumours and for cancer sites including at least 40 deaths. The model including gross household income was initially adjusted for household size (i.e. number of persons living in the same household) and marital status in a combined set of indicator variables. Subsequently, stage of disease (localised, regional metastasis, or distant metastasis) and smoking (current, former or never) were included in the multivariate models as a core set of covariates, regardless of their confounding effect. Other lifestyle or demographic variables such as body mass index as weight in kilos divided by height squared, level of physical activity, parity, use of postmenopausal hormone replacement therapy (HT) and hormonal contraceptives (HC), intake of alcohol, diet, and region of living were tentatively added to each site-specific model, and included whenever they changed the association of interest by at least 5%. Co-morbidity prior to diagnosis (prevalence of cardiovascular or coronary heart diseases, or poor perceived health) is included in the analysis of survival of all solid tumours by years of education regardless of this criterion. Tests for linear trend were carried out by the introduction of an ordinal variable obtained by assigning consecutive integers to the categories of education. The likelihood ratio test was applied to compare different models according to the impact of certain variables on mortality risk

## Results

Additional file 1 shows characteristics of the study population by years of education. Well-educated women were on the average younger and were less likely to be current smokers than the less educated. The distribution of tumour stage at diagnosis revealed a decreasing proportion of tumours with regional or distant metastasis with increasing years of education up to 13–16 years, whereas the highest educated women had a stage distribution similar to the middle educated (10–12 years of education). Additional file 2 shows that increasing gross household income was associated with a lower age at cohort enrolment and a smaller proportion of current smokers, and a decreasing proportion of women with advanced metastasis. Alcohol consumption increased both with increasing education and increasing household gross income. Prevalence of co-morbidity prior to diagnosis decreased with increasing SES. Additional file 3 gives the relative risks of cancer mortality by years of education. The age adjusted analysis shows a decreasing mortality with increasing education (RR = 0.65; 0.49–0.87 for women with more than 17 years of education compared to those with 7–9 years). The corresponding estimate for solid tumours only was slightly weaker (RR = 0.72; 0.52–0.98). Inclusion of stage modestly increased the risk difference between the highest and lowest education groups (RR = 0.68; 0.50–0.93). Co-morbidity did not alter the estimates, but further adjustment for smoking status reduced the mortality risk of the highest educated by 51% for all solid tumours (RR = 0.82; 0.60–1.12). The significant linear trend of declining mortality was evident for ovarian cancer in the site specific analyses (p = 0.04, RR = 0.51; 0.26–0.98 for women with 13–16 years of education compared to 7–9 years). On the other hand, mortality of colorectal cancer was observed to be increasing by years of education (p for linear trend = 0.04). For other solid tumours, inclusion of disease stage revealed a borderline significant effect for middle educated women (13–16 years) compared to the lowest educated (RR = 0.67; 0.46–0.99). For all solid tumours the associations declined into non-significance by further adjustment for smoking status, and for alcohol consumption in the analysis of colorectal cancer. The association between survival of all cancers and gross household income (see Additional file 4) showed a similar pattern as for education (RR = 0.68; 0.46–1.01 for the highest income group compared to the lowest). The observed linear trend in mortality risk of all cancers between income groups (p = 0.007) weakened by adjusting for household size and marital status (p = 0.05). Further adjustment for stage and smoking status offset the survival trend among all solid tumours.

The values of the likelihood ratio statistic displayed a greater variation in mortality risk by education (χ_3_^2 ^= 18.0, p = 0.0004) than by income (χ_4_^2 ^= 8.7, p = 0.07) after adjusting for age, when 3 329 individuals with information on both education and income were included. The value of the Spearman correlation coefficient between education and income was 0.40 (p < 0.0001).

## Discussion

Our study shows an inverse association between SES and age adjusted overall cancer mortality among cases diagnosed after study enrolment. The results were quite similar when different measures of SES, such as years of education or gross household income, were used. In the site-specific analyses by years of education the increased mortality risk among the low educated women was evident for ovarian cancer. The mortality risk among colorectal cancer patients increased with years of education.

The opportunity of taking into account a variety of potential confounders such as tumour stage, lifestyle before diagnosis (smoking, alcohol drinking, level of physical activity, diet, anthropometry), and prevalence of certain other diseases in the analyses of SES and cancer survival is a considerable advantage of the present study. As The NOWAC Study is prospective, the information on lifestyle and behaviour was collected before cancer diagnosis, and therefore, is not subject to recall bias, which is advantageous according to the understanding of causality. However, we did not have information on changes in behaviour after the time of diagnosis, which may have affected survival.

The measures of SES in our study are based on self-reported information. We have no access to register data on either education or income, which hampers a validation of the SES outcomes. Self-reported education often exceeds the number of years recorded in official statistics because the participants are likely to state both incomplete and informal training sessions. We believe that the self-reported level of income can be considered in accordance with official figures.

The estimates of cancer mortality risk among all cancer patients show a significantly reduced risk by increasing level of SES. We are aware that a part of the variation in risk may be explained by higher rates of cancers of poor prognosis (e. g. lung cancer) in individuals of low SES. In the analyses of survival by gross household income the observed linear trend in mortality risk of all cancers attenuated after adjusting for household size and marital status, whereas neither household size nor marital status affected educational differences in risk. Thus, the influence of these factors seems to be related to the importance of adjusting income measures for number of incomes in the household rather than adjusting for the potential psychosocial benefit of being married.

The values of the likelihood ratio statistic displayed a greater variation in mortality risk by education than by income. Lack of individual information on income among women in previous studies of cancer survival hampers any comparisons, but studies of other health outcomes suggest that the relative magnitude of each SES measure varies with outcome [10-15]. Different SES measures are dissimilarly related to underlying causal factors and cannot be used interchangeably [10,16]. A single measure of SES only partly explains the effect of another single measure [17]. In the present study the impact of smoking status on variation in survival is strong both for educational and income differences, but strongest for education. Education is probably the most adequate measure of SES, particularly among women; it applies to every adult individual, and is more stable over one's lifetime than income [18]. Adjustment for lifestyle or behavioural factors such as body mass index, level of physical activity, diet, reproductive history, use of HT or HC did not affect the socioeconomic variation, nor the impact of smoking on SES differences. In our analyses we have not been able to explain any portion of the effect of smoking by other factors, but according to overall survival we believe the effect of smoking is partly attributable to an excess of poor prognosis cancers among smokers. However, we observed an increased case mortality of specific cancer sites among current smokers, which supports that smoking prior to diagnosis may play a biological role in the progress of some cancer sites, but not all. Despite our ability to adjust for prevalence of certain diseases or poor perceived health at study enrolment, we cannot completely rule out the potential influence of co-morbidity on cancer survival according to smoking status.

The increased mortality risk in cancer patients of low SES groups observed in our study confirms findings by several previous studies [1,2,19-24]. However, our result of a slightly poorer prognosis of colorectal cancer among highly educated women is rarely supported [7]. Our finding of an improved survival by high SES for ovarian cancer does neither seem to be consistently evidenced [1,7,8].

Survival of cancer is influenced by several factors which can be classified into three groups: biological characteristics of the tumour (including stage at diagnosis), patient characteristics (e. g. lifestyle, health status), and treatment (quality of and access to health services) [8]. Consequently, SES differences in survival must originate from an unequal socioeconomic distribution of some of these factors. The predominant established prognostic factor of cancer survival is stage at diagnosis, as the classification of stage is derived from expected survival probability. According to SES differences in cancer survival, stage at diagnosis is an explaining factor often cited, but its influence varies by anatomic site and between populations [7]. A previous study following all cancer patients in Norway from 1960 to 1991 showed persisting differences in survival even after adjusting for stage [1]. The origin of social inequalities in stage distribution has also been discussed previously. Neither differences in timing of diagnosis nor differences in tumour aggressiveness have been evidenced to explain the variation [7,8], but a recent Danish study reports that patient characteristics predict delay in cancer diagnosis, among others in female smokers [25]. SES differences in cancer treatment have also been reported [7,8]. A few studies have considered the potential effect of psychosocial factors [1,26], and co-morbidity [27,28], and one study was able to control for smoking and alcohol consumption among men [29], finding a minor effect on crude survival. The results of our study question the distribution of tumour stage at diagnosis as a consistent mediator of SES variation in survival, but rather emphasize the influence of lifestyle factors such as smoking. Indeed, in our study, smoking status explained about 51% of mortality risk difference between the upper and lower educational groups after adjusting for age, stage, and co-morbidity prior to diagnosis.

## Conclusion

In summary, we found an overall negative socioeconomic gradient in cancer survival when SES is measured as years of education or gross household income. The contribution of stage at diagnosis on survival differences was inconsistent, whereas smoking status prior to diagnosis was an important predictive factor for survival. After adjustment for stage and smoking status survival differences according to both education and income turned into non-significance, and thus no significant variation is left for potential differences in treatment.

## Competing interests

The authors declare that they have no competing interests.

## Authors' contributions

TB is responsible for the drafting of the manuscript and performed the analyses. EW revised the manuscript. EL revised the manuscript, and is the PI of The NOWAC Study and the responsible for the collection of data. All authors read and approved the final manuscript.

## Pre-publication history

The pre-publication history for this paper can be accessed here:



## Supplementary Material

Additional file 1 **Characteristics of the incident cancer cases by years of education. The Norwegian Women and Cancer Study 1996–2005**. This file gives the means/percentages by years of education of all covariates included in the analyses Click here for file

Additional file 2 **Characteristics of the incident cancer cases by gross household income. The Norwegian Women and Cancer Study 1996–2005**. This file gives the means/percentages by gross household income of all covariates included in the analyses Click here for file

Additional file 3 **Relative risks (RR) with 95% confidence intervals (CI) of cancer mortality among patients diagnosed after study enrolment, by years of self-reported education. The Norwegian Women and Cancer Study 1996–2005**. The data provided represents the Cox regression analysis of relative mortality risks by categories of education. Whenever a variation in risk by SES was observed in the age adjusted analyses, potential confounding variables were added stepwise to the models Click here for file

Additional file 4 **Relative risks (RR) with 95% confidence intervals (CI) of cancer mortality among patients diagnosed after study enrolment, by self-reported gross household income. The Norwegian Women and Cancer Study 1996–2005**. The data provided represents the Cox regression analysis of relative mortality risks by categories of gross household income. Whenever a variation in risk by SES was observed in the age adjusted analyses, potential confounding variables were added stepwise to the models Click here for file
