# Supplementary material for: Socioeconomic differences in cancer survival: The Norwegian Women and Cancer Study
Source: BMC Public Health. 2009 Jun 8;9:178. doi: 10.1186/1471-2458-9-178 (PMC2702382; doi:10.1186/1471-2458-9-178)
Supplement: Additional file 1 — Characteristics of the incident cancer cases by years of education. The Norwegian Women and Cancer Study 1996–2005. This file gives the means/percentages by years of education of all covariates included in the analyses [file 1471-2458-9-178-S1.doc]

Characteristics of the incident cancer cases by years of education.

The Norwegian Women and Cancer Study 1996-2005.

| **Years of education** | | 7-9 | 10-12 | 13-16 | >=17 |
| --- | --- | --- | --- | --- | --- |
|  | N | % | % | % | % |
| Total | 3603 | 35.5 | 32.7 | 22.4 | 9.4 |
| Mean age ($\pm$SD) in years at cohort enrolment |  | 56.8  ($\pm$7.5) | 52.4  ($\pm$7.5) | 51.0  ($\pm$7.1) | 50.0  ($\pm$6.5) |
| Tumour stage at diagnosis |  |  |  |  |  |
| Localised | 1546 | 48.2 | 52.6 | 56.9 | 48.9 |
| Regional metastasis | 1063 | 37.2 | 34.3 | 33.2 | 38.9 |
| Distant metastasis | 383 | 14.6 | 13.1 | 9.9 | 12.2 |
| Co-morbidity |  |  |  |  |  |
| Yes | 585 | 22.5 | 13.2 | 14.0 | 8.3 |
| No | 3018 | 77.5 | 86.8 | 86.0 | 91.7 |
| Smoking status |  |  |  |  |  |
| Never | 1221 | 29.7 | 33.6 | 41.6 | 45.3 |
| Former | 1020 | 27.2 | 29.5 | 29.8 | 35.4 |
| Current | 1237 | 43.1 | 36.9 | 28.6 | 19.3 |
| Mean alcohol consumption in grams per day |  | 2.1 ($\pm$3.2) | 3.3 ($\pm$4.7) | 4.0 ($\pm$4.5) | 5.0 ($\pm$5.3) |
