# Supplementary material for: Socioeconomic differences in cancer survival: The Norwegian Women and Cancer Study
Source: BMC Public Health. 2009 Jun 8;9:178. doi: 10.1186/1471-2458-9-178 (PMC2702382; doi:10.1186/1471-2458-9-178)
Supplement: Additional file 2 — Characteristics of the incident cancer cases by gross household income. The Norwegian Women and Cancer Study 1996–2005. This file gives the means/percentages by gross household income of all covariates included in the analyses [file 1471-2458-9-178-S2.doc]

Characteristics of the incident cancer cases by gross household income.

The Norwegian Women and Cancer Study 1996-2005.

| **Gross household income** | | < 150 000 | 150 000 –  300 000 | 300 000 450 000 | 451 000 600 000 | > 600 000 |
| --- | --- | --- | --- | --- | --- | --- |
|  | N | % | % | % | % | % |
| Total | 3575 | 15.6 | 37.1 | 25.2 | 16.1 | 6.0 |
| Mean years of education |  | 9.2 ($\pm$3.0) | 10.9 ($\pm$3.2) | 11.6 ($\pm$3.2) | 13.3 ($\pm$3.3) | 14.4 (3.2) |
| Mean age ($\pm$SD) in years at cohort enrolment |  | 59.0  ($\pm$8.4) | 54.7  ($\pm$7.9) | 51.4  ($\pm$6.7) | 50.3  ($\pm$5.6) | 50.0  ($\pm$5.7) |
| Tumour stage at diagnosis |  |  |  |  |  |  |
| Localised | 1534 | 50.4 | 49.3 | 52.2 | 54.8 | 58.1 |
| Regional metastasis | 1051 | 35.3 | 36.2 | 35.8 | 34.7 | 30.5 |
| Distant metastasis | 387 | 14.3 | 14.6 | 12.0 | 10.5 | 11.5 |
| Co-morbidity |  |  |  |  |  |  |
| Yes | 575 | 27.5 | 18.2 | 14.1 | 7.0 | 6.5 |
| No | 3000 | 72.5 | 81.8 | 85.9 | 93.0 | 93.5 |
| Smoking status |  |  |  |  |  |  |
| Never | 1215 | 33.5 | 33.3 | 34.9 | 37.5 | 43.5 |
| Former | 1011 | 23.1 | 28.9 | 30.5 | 32.5 | 31.3 |
| Current | 1243 | 43.4 | 37.8 | 34.6 | 30.0 | 25.2 |
| Mean consumption of alcohol in grams per day |  | 1.8 (±3.5) | 2.7 (±3.6) | 3.4 (±4.2) | 4.3 (4.7) | 6.5 (7.2) |
