# Supplementary material for: Socioeconomic differences in cancer survival: The Norwegian Women and Cancer Study
Source: BMC Public Health. 2009 Jun 8;9:178. doi: 10.1186/1471-2458-9-178 (PMC2702382; doi:10.1186/1471-2458-9-178)
Supplement: Additional file 3 — Relative risks (RR) with 95% confidence intervals (CI) of cancer mortality among patients diagnosed after study enrolment, by years of self-reported education. The Norwegian Women and Cancer Study 1996–2005. The data provided represents the Cox regression analysis of relative mortality risks by categories of education. Whenever a variation in risk by SES was observed in the age adjusted analyses, potential confounding variables were added stepwise to the models [file 1471-2458-9-178-S3.doc]

Relative risks (RR) with 95 % confidence intervals (CI) of cancer mortality among patients diagnosed after study enrolment, by years of self-reported education. The Norwegian Women and Cancer Study 1996-2005.

| Cancer site | Adjustment | No of  deaths | Years of education | | | | p for linear trend |
| --- | --- | --- | --- | --- | --- | --- | --- |
|  |  |  | 7-9 | 10-12 | 13-16 | >=17 |  |
| All | Age | 842 | 1.00 (ref.) | 0.83 (0.71-0.98) | 0.70 (0.57-0.85) | 0.65 (0.49-0.87) | <0.0001 |
| All solid tumours | Age | 674 | 1.00 (ref.) | 0.82 (0.69-0.99) | 0.69 (0.56-0.86) | 0.72 (0.52-0.98) | 0.0007 |
|  | Age, stage |  | 1.00 (ref.) | 0.82 (0.68-0.98) | 0.72 (0.58-0.90) | 0.68 (0.50-0.93) | 0.0006 |
|  | Age, stage, co-morbidity |  | 1.00 (ref.) | 0.82 (0.69-0.99) | 0.73 (0.59-0.90) | 0.69 (0.50-0.94) | 0.0008 |
|  | Age, stage, co-morbidity, smoking status |  | 1.00 (ref.) | 0.89 (0.74-1.07) | 0.81 (0.65-1.01) | 0.82 (0.60-1.12) | 0.05 |
| Colon and rectum | Age | 123 | 1.00 (ref.) | 1.22 (0.78-1.90) | 1.55 (0.95-2.52) | 1.65 (0.88-3.07) | 0.04 |
|  | Age, stage |  | 1.00 (ref.) | 1.19 (0.76-1.85) | 1.60 (0.97-2.62) | 1.28 (0.68-2.38) | 0.14 |
|  | Age, stage, smoking status, alcohol consumption |  | 1.00 (ref.) | 1.18 (0.75-1.84) | 1.41 (0.83-2.41) | 1.24 (0.65-2.36) | 0.28 |
| Lung | Age | 177 | 1.00 (ref.) | 0.88 (0.63-1.24) | 1.18 (0.69-1.99) | 1.00 (0.36-2.74) | 0.95 |
| Breast | Age | 112 | 1.00 (ref.) | 1.34 (0.82-2.18) | 1.13 (0.66-1.92) | 1.37 (0.71-2.64) | 0.48 |
| Ovary | Age |  | 1.00 (ref.) | 0.75 (0.43-1.29) | 0.51 (0.26-0.98) | 0.55 (0.21-1.45) | 0.04 |
|  | Age, stage |  | 1.00 (ref.) | 0.70 (0.41-1.21) | 0.56 (0.29-1.09) | 0.50 (0.19-1.32) | 0.05 |
|  | Age, stage, smoking status |  | 1.00 (ref.) | 0.71 (0.41-1.23) | 0.56 (0.28-1.12) | 0.55 (0.21-1.48) | 0.08 |
| Other solid tumours | Age | 208 | 1.00 (ref.) | 0.79 (0.57-1.10) | 0.75 (0.51-1.11) | 0.87 (0.48-1.58) | 0.20 |
|  | Age, stage |  | 1.00 (ref.) | 0.95 (0.68-1.32) | 0.67 (0.46-0.99) | 0.83 (0.46-1.51) | 0.09 |
|  | Age, stage, smoking status |  | 1.00 (ref.) | 1.05 (0.75-1.46) | 0.79 (0.53-1.17) | 0.97 (0.53-1.78) | 0.41 |
