# Supplementary material for: Socioeconomic differences in cancer survival: The Norwegian Women and Cancer Study
Source: BMC Public Health. 2009 Jun 8;9:178. doi: 10.1186/1471-2458-9-178 (PMC2702382; doi:10.1186/1471-2458-9-178)
Supplement: Additional file 4 — Relative risks (RR) with 95% confidence intervals (CI) of cancer mortality among patients diagnosed after study enrolment, by self-reported gross household income. The Norwegian Women and Cancer Study 1996–2005. The data provided represents the Cox regression analysis of relative mortality risks by categories of gross household income. Whenever a variation in risk by SES was observed in the age adjusted analyses, potential confounding variables were added stepwise to the models [file 1471-2458-9-178-S4.doc]

Relative risks (RR) with 95 % confidence intervals (CI) of cancer mortality among patients diagnosed after study enrolment, by self-reported gross household income. The Norwegian Women and Cancer Study 1996-2005.

| Cancer site | Adjustment | No of deaths | Gross household income in Norwegian Crowns | | | | | P for linear trend |
| --- | --- | --- | --- | --- | --- | --- | --- | --- |
|  | | | < 150 000 | 151 000-300 000 | 301 000-450 000 | 451 000-600 000 | > 600 000 |  |
| All | Age | 814 | 1.00 (ref.) | 0.95 (0.78-1.15) | 0.82 (0.66-1.03) | 0.78 (0.60-1.01) | 0.68 (0.46-1.01) | 0.007 |
|  | Age, household size, marital status |  | 1.00 (ref.) | 0.97 (0.80-1.18) | 0.87 (0.68-1.10) | 0.82 (0.62-1.09) | 0.73 (0.49-1.09) | 0.05 |
| All solid tumours | Age | 652 | 1.00 (ref.) | 0.99 (0.80-1.22) | 0.83 (0.65-1.07) | 0.76 (0.57-1.02) | 0.75 (0.49-1.15) | 0.02 |
|  | Age, household size, marital status, stage |  | 1.00 (ref.) | 0.84 (0.67-1.04) | 0.83 (0.64-1.09) | 0.79 (0.58-1.07) | 0.75 (0.49-1.16) | 0.14 |
|  | Age, household size, marital status, stage, smoking status |  | 1.00 (ref.) | 0.87 (0.70-1.08) | 0.90 (0.69-1.17) | 0.88 (0.65-1.20) | 0.85 (0.55-1.32) | 0.51 |
| Colon and rectum | Age | 133 | 1.00 (ref.) | 0.87 (0.54-1.40) | 0.73 (0.41-1.32) | 1.00 (0.53-1.88) | 1.13 (0.50-2.53) | 0.87 |
| Lung | Age | 177 | 1.00 (ref.) | 1.34 (0.85-2.12) | 1.25 (0.74-2.10) | 1.66 (0.94-2.92) | 1.20 (0.35-4.09) | 0.22 |
| Breast | Age | 106 | 1.00 (ref.) | 0.97 (0.52-1.81) | 0.91 (0.46-1.78) | 0.88 (0.43-1.82) | 0.95 (0.37-2.43) | 0.75 |
| Ovary | Age | 79 | 1.00 (ref.) | 0.70 (0.38-1.31) | 0.58 (0.29-1.15) | 0.58 (0.29-1.15) | 0.96 (0.22-4.24) | 0.24 |
| Other solid tumours | Age | 202 | 1.00 (ref.) | 0.96 (0.67-1.37) | 0.94 (0.61-1.44) | 0.61 (0.35-1.08) | 0.78 (0.36-1.67) | 0.14 |
